# A Globally Guided Dual-Arm Reactive Motion Controller for Coordinated Self-Handover in a Confined Domestic Environment

**DOI:** 10.3390/biomimetics9100629

**Published:** 2024-10-16

**Authors:** Zihang Geng, Zhiyuan Yang, Wei Xu, Weichao Guo, Xinjun Sheng

**Affiliations:** 1 State Key Laboratory of Mechanical System and Vibration, School of Mechanical Engineering, Shanghai Jiao Tong University, Shanghai 200240, China; gengzihang@sjtu.edu.cn (Z.G.); jonathan-y@sjtu.edu.cn (Z.Y.); xu.wei@sjtu.edu.cn (W.X.); 2 Meta Robotics Institute, Shanghai Jiao Tong University, Shanghai 200240, China

**Keywords:** motion planning, dual-arm coordination, humanoid robot, global planning, reactive control, collision avoidance

## Abstract

Future humanoid robots will be widely deployed in our daily lives. Motion planning and control in an unstructured, confined, and human-centered environment utilizing dexterity and a cooperative ability of dual-arm robots is still an open issue. We propose a globally guided dual-arm reactive motion controller (GGDRC) that combines the strengths of global planning and reactive methods. In this framework, a global planner module with a prospective task horizon provides feasible guidance in a Cartesian space, and a local reactive controller module addresses real-time collision avoidance and coordinated task constraints through the exploitation of dual-arm redundancy. GGDRC extends the start-of-the-art optimization-based reactive method for motion-restricted dynamic scenarios requiring dual-arm cooperation. We design a pick–handover–place task to compare the performances of these two methods. Results demonstrate that GGDRC exhibits accurate collision avoidance precision (5 mm) and a high success rate (84.5%).

## 1. Introduction

Humanoid robots become a great choice for the integration of embodied intelligence [1], and exhibit greater interactivity and social acceptance than traditional industrial and service robots. Benefiting from anthropomorphic hardware design [2,3] and the continuous learning of human locomotor abilities, these robots will become a new intelligent workforce that can adopt to human-centered environments, use existing tools and facilities, and assist or replace humans in complex tasks. Using humanoid robots in industrial manufacturing is a very promising trend. Tasks like large-volume object transferring [4], automobile parts assembly [5], and battery sorting have been tested in a real factory scenario. In the field of rehabilitation and elderly care, humanoid robots can support the elderly with motor disabilities and assist in standing and walking [6]. As for home services, future humanoid robots will play the role of servant and free people from tedious chores like folding clothes, cleaning the kitchen [7], and organizing items [8].

**Figure 1:**
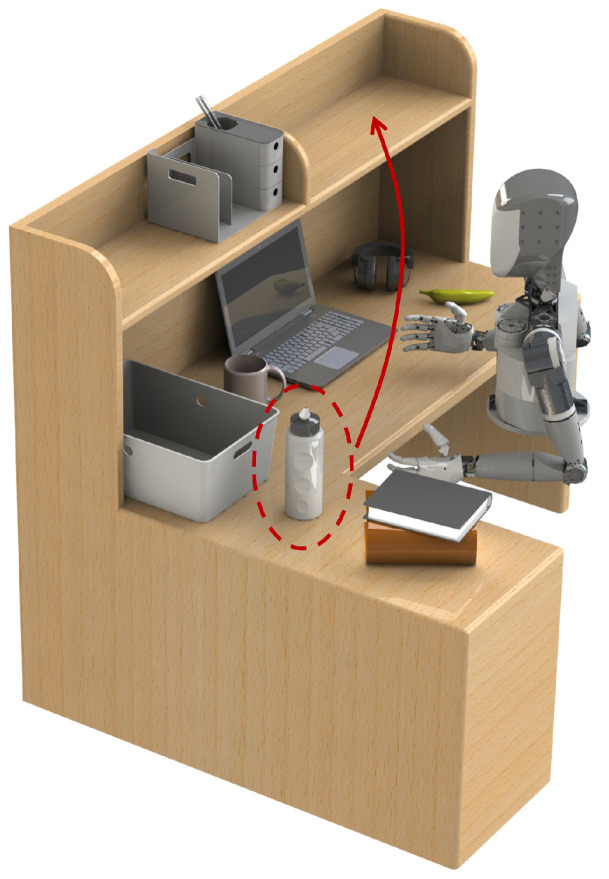
Desktop organizing task. In this cluttered scenario, a dual-arm humanoid robot picks up the water bottle and puts it on the shelf.

These dual-arm robots, compared with single-arm ones, have a much larger workspace and show more versatility and dexterity that comes from the coordination of two manipulators [9,10,11]. They are capable of performing complex tasks, as mentioned previously, that are not possible with a single arm. Nevertheless, high degrees of freedom and the redundant nature of dual-arm humanoid robots extremely increase the complexity of modeling, motion planning, and control. The spatial and temporal coordination [12] and close-chain motion constraints [13] of two arms pose additional challenges for bimanual manipulation. Considering the human-like configuration, self-collision avoidance is required because of the overlapped workspace of two arms [14,15]. Moreover, future humanoid robots will be employed in unstructured and motion-restricted environments, which also exhibit volatile properties due to human co-existence. Like the desktop organizing scenario shown in Figure 1, we focus on the dual-arm handover task with coordination constraints within a confined space, taking into account possible dynamic changes.

The perception–planning–implementation architecture is widely used in most classical methods. Sample-based planners, like RRT family, are typical representatives that explore the joint configuration space to obtain a collision-free and physically practical trajectory [16]. Although the completeness of a resulting trajectory is guaranteed, these planners encounter great computational difficulties in terms of high-dimensional problems. Apart from that, dual-arm coordinated tasks require sampling in constrained manifolds [17]. How to define this unconventional sample space and obtain feasible dual-arm trajectories remains a problem. Another category of global planning approaches is based on trajectory optimization [18,19,20,21,22]. These methods construct the optimization cost function and constraints from sub-objectives like collision avoidance, robot physical limits, trajectory smoothness, and other task-specific conditions. It is then solved with algorithms like sequential convex optimization [18] or an iterative linear quadratic regulator (iLQR) [22]. The strength of these methods is that dual-arm coordination constraints can be integrated into the optimization problem effortlessly. However, when handling tasks with high degrees of freedom systems or long time spans, the global optimization problem is also very time-consuming. Researchers usually utilize prior knowledge to expedite the process. In [20], based on solved tasks and previously trained deep learning networks, the optimization problem is warmed up with an initialization value close to the optimal solution. Additionally, in [21], a knowledge database is constructed for skill digitization to accelerate the trajectory planning of a ping-pong robot. Nonetheless, all methods above cannot meet the demand for reactiveness in dynamic and confined environments; consequently, they are inappropriate to be applied in scenarios tightly coupled with human activities.

Instead of planning a whole trajectory, reactive methods are built to handle dynamic tasks. This type of methods focuses on motion planning in a short time horizon and generates instantaneous control commands. An artificial potential field is a commonly used approach that finds a path by introducing virtual repulsive forces between a robot and obstacles and an attractive force to the goal configuration. Meanwhile, in [23], a quadratic optimization problem is constructed considering obstacle avoidance and robot differential kinematics, whose solution is used as the momentary velocity control input. This method also optimizes robot manipulability to improve the success rate of dynamic obstacle avoidance. Additionally, there are online re-planning methods like Model Predictive Control (MPC), which optimizes robot motion in a short period according to task-specific criteria and adopts the solution in the first time step. With a similar idea, [24] combines MPC with a nonprehensile manipulation model and realizes flying obstacle avoidance while balancing stacked objects. However, all these reactive methods are hindered by their limited planning horizon. The myopic nature only leads to locally feasible solutions. In clustered and confined environments, especially under the circumstances where there are conflicting task sub-objectives like collision avoidance and goal approaching, these methods tend to get trapped in a local optimum. Recently, a hierarchical motion generation method is proposed, which is essentially a weighted product of reactive expert policies [25]. With the stochastic optimization of optimal weights over the task horizon, it avoids local optima and generates feasible plans in a cluttered environment. Nevertheless, this method does not take into account the dual-arm cooperation in the task. In fact, dual-arm coordinated motion is mostly addressed by the model-based design or learning from the demonstration for a specific task [26,27].

To address the challenges of humanoid robots operating in a dynamic confined environment with dual-arm coordinated constraints (like desktop cleaning tasks), we designed the globally guided dual-arm reactive motion controller (GGDRC). This framework leverages the strengths of global prospective planning and local reactive methods. It is inspired by the human motion strategy: a coarse path of the manipulated object is rapidly planned in advance, and then during the real movement process, the motion behavior of arms and hands is adjusted in real time according to the interaction information with the environment. The long-horizon task information provides feasible guidance, leading the robot away from the local minimum. With the conversion of optimization space and reasonable dimension reduction, the computational efficiency of an object-oriented global planner is greatly improved. The local reactive controller, in the meanwhile, provides high-frequency control capability to deal with the dynamic changes. It takes full advantage of the versatility and dexterity of redundant manipulators to address real-time collision avoidance and coordinated task constraints. An accurate collision avoidance precision is realized by our modified velocity damper method.

We summarize our main contributions as follows:

(1) A bi-level framework GGDRC combining global planning and reactive control for humanoid robots performing dual-arm coordinated tasks in dynamic motion-restricted environments;
(2) A fast global planner that generates object-oriented reference trajectories in a Cartesian space with sequential quadratic programming;
(3) A reactive motion controller for redundant robots that achieves dual-arm coordination and precise collision avoidance.

## 2. Materials and Methods

**Figure 2:**
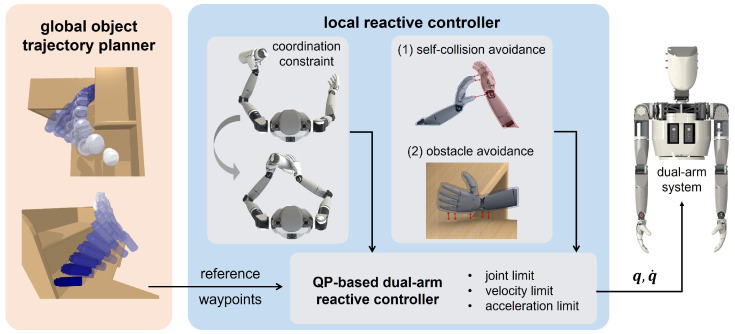
Framework of a globally guided dual-arm reactive controller (GGDRC). This framework consists of two modules: (**left**) global object trajectory planner for reference waypoints over the task horizon and (**right**) QP-based local reactive controller for dual-arm motion coordination and obstacle avoidance exploiting kinematic redundancy.

The framework is illustrated in Figure 2. It can be roughly divided into two modules: global object trajectory planner and local reactive motion controller. The global planner is designed to provide reference trajectory points over the task horizon and improve the feasibility of the dual-arm motion. To get rid of the low efficiency of traditional global planning methods, our global planner focuses on the target object trajectory in a Cartesian space and uses sparser reference waypoints. In this fashion, we can significantly reduce the time consumption while still maintaining the validity of the global guidance. The local reactive motion controller based on quadratic programming (QP) exploits the redundancy of a dual-arm robot that has high degrees of freedom, and addresses real-time environment collision and robot self-collision avoidance problems. The task-specific dual-arm coordination constraint based on the relative Jacobian of the robot is also integrated into the local controller. With a lot of well-developed algorithms for QP, this controller can be solved within 10 ms and, hence, has the capability to deal with dynamic obstacles. The implementation details of each module will be described in the rest of this section.

### 2.1. Dual-Arm Humanoid Robot Model and Kinematic Analysis

**Figure 3:**
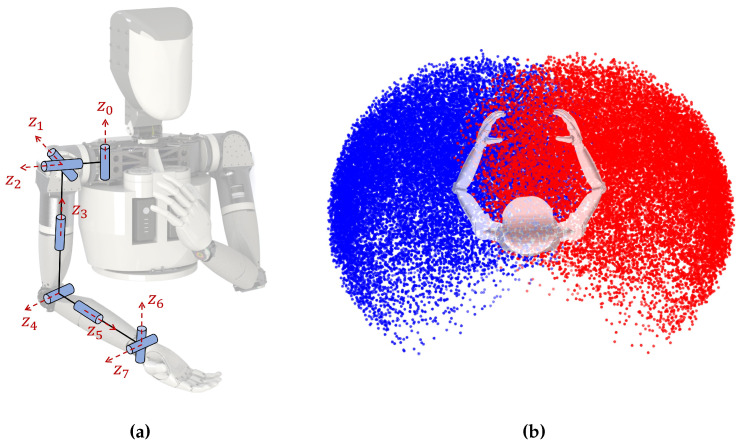
Dual-arm model and overlapped workspace: (**a**) dual-arm model: z0−z7 denote rotation axes of the joints; (**b**) overlapped workspace: blue area denotes the workspace of left arm and red of the right arm.

This work is based on our dual-arm humanoid robot system. Each arm is designed to have 8 degrees of freedom with an anthropomorphic configuration. Unlike other humanoid arms using a 3-DOF shoulder, we have added an extra degree of freedom at the root of the shoulder to mimic the protraction of the human scapula. As shown in Figure 3a, this arm has a 4-DOF shoulder, a 1-DOF elbow, and a 3-DOF wrist. The main difference between a dual-arm system and a single-arm one is the capacity of cooperative manipulation, which takes place at the overlapped workspace of the arms demonstrated in Figure 3b.

**Figure 4:**
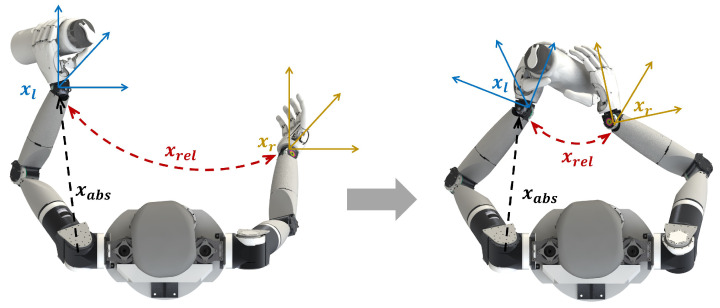
Dual-arm motion decomposition in a self-handover task. In independent mode, $x_{l}$ and $x_{r}$ denote the left EE motion and the right EE motion, respectively. Meanwhile, in coordinated mode, $x_{rel}$ is the relative motion between two end effectors and $x_{abs}$ is the absolute object motion simplified as $x_{l}$.

The motion planning of a dual-arm robot is inherently more complicated due to the high redundancy and overlapped workspace. Depending on whether two arms move for a unified target, a dual-arm movement can be classified as an independent motion or a coordinated motion. In the former category, two arms have separate goals, and the motion of both end effectors should be specified by the controller. However, there is always an association between the two arms as self-collision avoidance needs to be taken into consideration all the time. As for the coordinated motion, we follow the convention of the extended cooperative task space introduced by [28]. The coordinated motion is converted to the relative motion of two arms plus the absolute task motion. Figure 4 demonstrates the components of a dual-arm motion in a self-handover task: $x_{rel}$ denotes the relative motion of two end effectors, which also specifies the cooperative task constraint, and $x_{abs}$ denotes the absolute motion of the manipulated object. When the left hand grasps the object, we simplify $x_{abs}$ using the end effector (EE) motion of the left arm since a fixed offset from the left EE to the object is maintained by the grasp constraint. Our controller applies to both of the two categories, which will be described in a later section.

Similar to the single-arm case in which the desired EE velocity in the task space can be converted to a robot configuration space by the inverse of kinematic Jacobian (or pseudo-inverse for a redundant manipulator), we describe the dual-arm coordinated motion as the relative EE velocity based on the relative Jacobian as follows:

Let $\sum_{l}$, $\sum_{r}$, and $\sum_{b}$ represent the frames fixed to the left and right EE and the robot base link. The subsequent upper-left or lower-right corner label i/j represents one of the three aforementioned coordinate frames. pji and Rji denote the rotation and translation of the frame $\sum_{j}$ with respect to $\sum_{i}$. Additionally, p˙ji and ωji are the corresponding velocities. Let $J_{i}={\left[J_{i_{v}}^{T},\;J_{i_{\omega }}^{T}\right]}^{T}$ in which i=l/r denotes the left- or right-arm Jacobian mapping joint space velocities ${\dot{q}}_{i}$ to EE velocity vib=[p˙iT,ωiT]T, i.e., vib=Jiq˙i. According to the rigid-body kinematics [29], the relative velocity of the right EE with respect to the left EE is calculated as follows:

(1) p˙rl=−lRbJlvq˙l+(−lRbJlωq˙l)×prb+RblJrvq˙r

(2) ωrl=−RblJlωq˙l+RblJrωq˙r

The relative velocity can be written in the following matrix form:

(3) vrel=p˙rlωrl=I3−[prb]×0I3−Rbl00−RblJlvJlωRbl00RblJrvJrωq˙lq˙r

We define the dual-arm joint space velocity $\dot{q}={\left[{\dot{q}}_{l}^{T},\;{\dot{q}}_{r}^{T}\right]}^{T}$ and the relative Jacobian $J_{rel}$ as follows:

(4) Jrel=I3−[prb]×0I3−Rbl00−RblJlRbl00RblJr

Hence, the task-specific dual-arm coordinated constraint can be described as the relative velocity based on the relative Jacobian, as follows:

(5) $$v_{rel}=J_{rel}\;\dot{q}$$

A humanoid robot exhibits great potential for more dexterous manipulation due to dual effectors and high degrees of freedom. Nevertheless, there are more singular configurations because of the hardware structure of the robot. Without an effective redundancy resolution, we may obtain a disappointing joint configuration that takes more effort to make a target movement. Manipulability is an important criterion describing the moving capability of a manipulator at some joint configuration. In this paper, we use the dual-arm relative manipulability ellipsoid as an extension of the single-arm case [30]. It is a geometrical visualization of the relative velocities whose corresponding joint space velocities $\dot{q}$ satisfy ${\dot{q}}^{T}\dot{q}=1$ [31]. Therefore, the mathematical representation of the ellipsoid is as follows:

(6) $$v_{rel}{\left(J_{rel}J_{rel}^{T}\right)}^{-1}v_{rel}=1$$

Then the corresponding dual-arm relative manipulability $\mu_{rel}$, proportional to the volume of the relative manipulability ellipsoid, is defined based on the relative Jacobian $J_{rel}$, as follows:

(7) $$\mu_{rel}=\sqrt{det(J_{rel}J_{rel}^{T})}$$

Our local controller takes the optimization of $\mu_{rel}$ as part of the objective function to avoid singular configurations and improve manipulability, which plays a more important role in the non-stationary environment.

### 2.2. Object-Oriented Global Planner

A widely used optimization-based method for global motion planning is sequential convex optimization. It takes a full joint space trajectory over the task horizon as the optimized variable, and solves the original non-convex optimization problem by iteratively simplifying it as a convex sub-problem [18,19]. Every sub-problem is a valid approximation of the original problem at the current iteration. The sub-problems are transformed to quadratic programs by converting the unfeasible constraints into penalties. That is why this method is called sequential quadratic programming (SQP). However, the conventional method is not suitable for our dual-arm robot. Because of the 16 DOFs and the clustered environment, it is hard to generate the whole trajectory in a short period of time.

Hence, we propose a fast object-oriented global reference trajectory planner. This planner shows great enhancement on the computational efficiency due to two main improvements.

First, we make an object-oriented planning to reduce the motion dimension. In the original optimization process, all 16 DOFs in the joint space need to be taken into account at each time step. However, in our dual-module framework, the global planner focuses on generating a feasible path of a target object in the task space, which is similar to the logic of human movement. The optimized component at each time step can be consequentially reduced to the 6-dimension pose of the target object. Moreover, in most object-transfer tasks, the position change plays a dominant role. Hence, we can even make an interpolation between start and goal orientations and only optimize the object positions.

Second, we reduce the waypoint density. The conventional SQP method relies on a dense trajectory or utilizes the convex envelop of the link movement to ensure that no collision occurs between adjacent time steps. Instead of addressing the problem in one shot, we integrate the meticulous robot collision constraints into a local reactive controller and emphasize the guiding rule in global planning. Therefore, a sparser set of continuous waypoints is sufficient to provide crucial motion reference, which further reduces the dimension of the entire optimization problem.

The trust region method is also adopted to maintain the validity of approximation like TrajOpt [18]. The object-oriented global reference trajectory planner is summarized in Algorithm 1.
**Algorithm 1** Object-Oriented Global Planner**Require:** 
$T_{start}$ and $T_{goal}$ are the start and goal pose of the object, k+1 is the number of waypoints, $d_{safe}$ is the preferred safe distance for collision, $S_{max}$ is the maximum step between adjacent waypoints, $\lambda_{p}$ is the weight coefficient for penalties, $\epsilon_{f}$ is the validity coefficient for linear approximation, $\epsilon_{x}$ is the convergence threshold, α>1 and 0<β<1 are trust region scaling factors**Ensure:** 
$x={\left[x_{0}^{T},x_{1}^{T},\cdots ,x_{k}^{T}\right]}^{T}$ is the object trajectory and $x_{j}$ for j=0,1,⋯,k is the position of *j*-th the waypoint 1: $x^{\left(0\right)}$← interpolation between $T_{start}$ and $T_{goal}$ 2: tr← initialize trust region 3: i←0 4: **while** True **do**             ▹ construct sub-optimization problem 5:    **for** j=0,1,⋯,k **do** 6:        $d(x^{\left(i\right)})$← computation of minimum distance 7:        $\hat{d}\left(x^{\left(i+1\right)}\right)\;\leftarrow d\left(x^{\left(i\right)}\right)+J({\hat{x}}^{\left(i\right)})\left(x^{\left(i+1\right)}-x^{\left(i\right)}\right)$ 8:        $t_{j}\geq 0$← add slack variables for penalty 9:        $d_{safe}-sd\left(x^{\left(i+1\right)}\right)\leq t_{j}$← collision constraint for QP10:    **end for**11:    $Cx^{\left(i+1\right)}\leq S_{max}$← limit the maximum distance between adjacent waypoints12:    $Ax^{\left(i+1\right)}=B$← set the start and goal pose w.r.t. $T_{start}$ and $T_{goal}$13:    $x^{\left(i+1\right)}\;\leftarrow$$argmin\;\hat{f}\left(x\right)=\sum_{j=0}^{k-1}\left|\right|x_{j+1}-x_{j}{\left|\right|}^{2}+\lambda_{p}\sum_{j=0}^{k}t_{j}$14:                  s.t. all constraints above and trust region $|x^{\left(i+1\right)}-x^{\left(i\right)}|\leq tr$15:    **if** $|x^{i+1}-x^{i}|\leq \epsilon_{x}$** then**                  ▹ check convergence16:        break17:    **end if**18:    i←i+1                    ▹ accept the current iteration19: **end while**20: $x^{*}\leftarrow x^{\left(i+1\right)}$ finish the iteration and adopt the current trajectory

### 2.3. QP-Based Local Reactive Controller

A generic quadratic programming problem takes the following form [32]:

(8) $$\begin{array}{cc} \underset{x}{min}\;f\left(x\right) & =\frac{1}{2}x^{T}Qx+l^{T}x \end{array}$$

(9) $$\begin{array}{cc} s.t.\;Ax & =B \end{array}$$

(10) $$\begin{array}{cc} Cx & \leq D \end{array}$$

where $x\in R^{n}$ is the n dimensional variable, $Q\in R^{n\times n}$ is the symmetric cost matrix, $l\in R^{n}$ is the linear part of the objective function, $A\in R^{m_{1}\times n}$ denotes the linear equality matrix, and $C\in R^{m_{2}\times n}$ denotes the linear inequality matrix.

Our local reactive controller is constructed based on the QP architecture for the following reasons: (a) the dual-arm coordinated motion and robot mechanical limits can be represented as linear constraints in the form of Equations (9) and (10), and (b) QP is computationally efficient, which is suitable for real-time applications. There are a lot of algorithms for a QP problem. In this paper, we use the Python library *qpsolvers*. The rest of this section describes how we integrate every core aspect of a dual-arm motion into the objective function or the linear constraint of QP.

#### 2.3.1. Dual-Arm Motion Constraints

Knowing the relative Jacobian $J_{rel}$ and the simplified absolute task motion, the dual-arm coordinated motion constraints can be expressed as follows:

(11) $$\left\{ \begin{array}{c} J_{rel}\dot{q}=v_{rel}\\ J_{abs}\dot{q}=v_{abs} \end{array}\right.$$

where $J_{abs}=J_{l/r}$. Additionally, an independent motion is easily represented with the individual arm Jacobians.

(12) $$\left\{ \begin{array}{c} J_{l}{\dot{q}}_{l}=v_{l}\\ J_{r}{\dot{q}}_{r}=v_{r} \end{array}\right.$$

In both cases, the velocities from the right side of the equation depend on current task requirements. In our GGDRC framework, they are determined by the reference waypoints from the global planner.

Note that the global guidance is not guaranteed to be practical as the robot mechanical limits are not considered in the global planning module. Under some circumstances where the robot is at a high risk colliding with the environment, the collision avoidance is supposed to be set at the first priority. Therefore, we relax these velocities in Equations (11) and (12) with slack variables $\delta \in R^{6}$ and $\eta \in R^{6}$. Then we minimize these slack variables to always encourage the consistency of the current robot motion and the global reference waypoints [23,32]. As a result, the relaxed dual-arm motion constraints are as follows:

(13) $$\left\{ \begin{array}{c} J_{rel}\dot{q}+\delta =v_{rel}\\ J_{abs}\dot{q}+\eta =v_{abs} \end{array}\right.$$

(14) $$\left\{ \begin{array}{c} J_{l}{\dot{q}}_{l}+\delta =v_{l}\\ J_{r}{\dot{q}}_{r}+\eta =v_{r} \end{array}\right.$$

#### 2.3.2. Collision Avoidance Constraints

**Figure 5:**
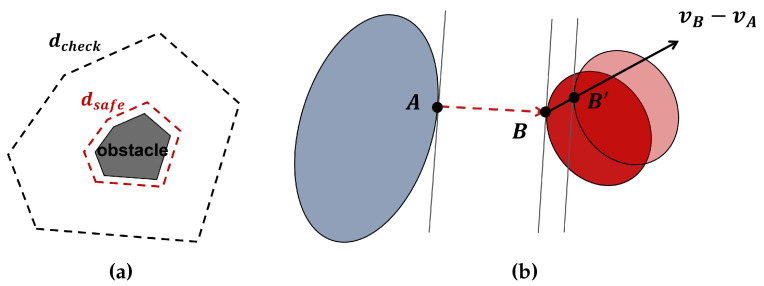
The velocity damper (VD) method. (**a**) VD is activated when the minimum distance $d_{min}$ is less than the check distance $d_{check}$. Then $d_{safe}$ is the ideal safe boundary. (**b**) Illustration of the VD applying to convex geometries. The blue and red ellipses denote the current positions on which *A* and *B* are the witness points. The translucent red ellipse denotes the relative position in next time step on which $B^{\prime }$ is the point corresponding to *B*. The relative velocity $v_{B}-v_{A}$ will lead two ellipses away from each other.

Velocity damper (VD) is a reactive method for robot collision avoidance [33]. The core idea is to restrict the velocity of the point on the robot based on its distance to the obstacle. In practice, every link of the robot is simplified as a convex geometry and environment objects are represented with a set of convex hulls. Like in Figure 5a, there is a check distance $d_{check}$ for the activation of VD and a safe distance $d_{safe}$ to limit the minimum allowable boundary.

As shown in Figure 5b, *A* and *B* are the witness points (closest points) on two convex geometries. Let $vd=\overset{⟶}{n_{AB}}\cdot \left(v_{B}-v_{A}\right)$ in which $\overset{⟶}{n_{AB}}$ is the unit direction vector from the witness point *A* to *B*. If vd>0, the distance between two points will increase in next time step; otherwise, it will decrease.

There are three main phases, which are determined by the minimum distance $d_{min}$ between two convex geometries. In phase I, when $d_{min}>d_{check}$, there is no limit on the velocity at this time step. In phase II, when $d_{check}\geq d_{min}>d_{safe}$, an inequality constraint is set on vd, which denotes the relative translational velocity of the witness points on two geometries, as follows:

(15) $$vd\geq -\xi_{v}\frac{d_{min}-d_{safe}}{d_{check}-d_{safe}}$$

where $\xi_{v}$ is the damping coefficient [12,23]. With this constraint, the maximum approaching velocity is damped when two geometries are getting close, while there is no limit when they are moving away. In phase III, when $d_{min}\leq d_{safe}$, a constraint vd>0 is made to make the distance of two witness points increase.

**Figure 6:**
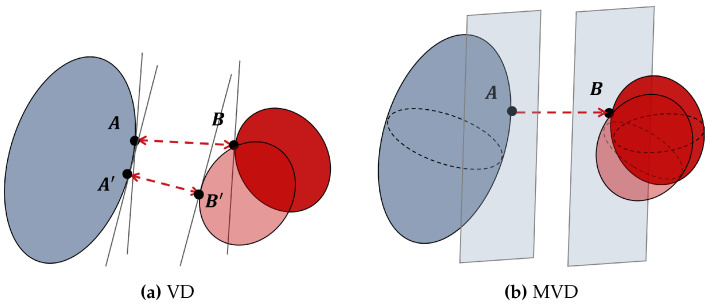
Comparison between VD and MVD at a safe boundary. (**a**) In this 2d VD example, witness points change (from *A* and *B* to *A’* and *B’*) because of the relative rotation. The minimum distance decreases in spite of a velocity damper applied to *A* and *B*. (**b**) In this 3d MVD example, the witness points are consistent. Combining translation and rotation dampers, two geometries are kept away from the separate plans.

However, the VD method is only able to increase the distance between the witness points, since only the velocity of one point is damped on each convex geometry. If there are both relative translation and rotation, the witness points in the next time step may not be consistent. This will lead to a situation where the minimum distance of two convex geometries actually decreases even though the origin witness points move away or remain relatively static. Figure 6a is an illustration in 2d space.

Therefore, we have proposed a new modified velocity damper (MVD) approach. It not only restricts the relative translation of witness points, but also limits the relative rotation of convex geometries by adding an extra rotation damper on directions orthogonal to the normal vector $\overset{⟶}{n_{AB}}$. Figure 6b is an illustration of MVD in 3d space. We define ωd as the restricted component of rotational velocity.

(16) $$\omega d=\left(\omega_{B}-\omega_{A}\right)-\left[\overset{⟶}{n_{AB}}\cdot \left(\omega_{B}-\omega_{A}\right)\right]\overset{⟶}{n_{AB}}$$

The infinity norm of ωd is adopted to construct the linear constraints in a similar fashion, as follows:

(17) $${\left|\omega d\right|}_{\infty }\leq \xi_{\omega }\frac{d_{min}-d_{safe}}{d_{check}-d_{safe}}$$

where $\xi_{\omega }$ is the rotational damping coefficient. With this constraint, the relative rotation that may cause changes on witness points is damped. Therefore, in the next time step, the minimum distance $d_{min}$ will not have much difference from the distance between the current witness points A and B. Equations (15) and (17) fully define our MVD collision constraints in phase II. Additionally, two constraints, ${\left|\omega d\right|}_{\infty }=0$ and vd>0, are combined to force the distance of two geometries to increase in phase III.

For robot links, the translational velocity of witness points and the rotational velocity of the corresponding links can be calculated with the truncated arm Jacobian ${\hat{J}}_{i}$, which is only related to joints 0 to *i*. For environment objects, the velocities are obtained from sensor measurements. With stricter collision constraints, the MVD method is supposed to perform better in a confined environment requiring a smaller safe distance.

#### 2.3.3. Robot Physical Constraints

Robot physical constraints such as joint angle limits and joint velocity limits can be easily wrapped into linear inequality constraints. Let $q^{-}$ and $q^{+}$ be the lower and upper angular limits and ${\dot{q}}^{-}$ and ${\dot{q}}^{+}$ be the velocity lower and upper limits, as follows:

(18) $${\dot{q}}^{-}\leq \dot{q}\leq {\dot{q}}^{+}$$

(19) $$q^{-}\leq q\leq q^{+}$$

where a joint angle is integrated by velocities $q=q_{0}+\dot{q}\cdot ▵t$.

#### 2.3.4. Objective Function of QP

To encourage smooth and minimum-length trajectories in a joint space, we use the squared joint velocities as the first sub-objective, as follows:

(20) $$f_{1}\left(q\right)=\frac{1}{2}{\dot{q}}^{T}W_{q}\dot{q}$$

where $W_{q}\in R^{16\times 16}$ is the diagonal weight matrix for different joints. Since the joints closer to the torso of the humanoid robot have a greater impact on the EE movement especially on translation, we assign a lower weight at the related index of $W_{q}$.

To obtain a joint configuration competent for highly demanding manipulation tasks, we need to incorporate the optimization of manipulability into the objective function [22]. To be compatible with the QP architecture and with Equation (7), the derivative of robot manipulability μ∈R to joint angles $q\in R^{n}$ is calculated as follows:

(21) $$\frac{d\mu }{dq}=\frac{d\sqrt{det(JJ^{T})}}{dq}={\left[\begin{array}{cccc} \frac{\partial \mu }{\partial q_{1}} & \frac{\partial \mu }{\partial q_{2}} & \cdots & \frac{\partial \mu }{\partial q_{n}} \end{array}\right]}^{T}$$

where *J* is the robot Jacobian and $\frac{\partial \mu }{\partial q_{i}}$ is the derivative of the manipulability with respect to the *i*-th joint angle. We take the element of the vector $\frac{\partial \mu }{\partial q}$, simply it with the chain rule of matrix derivation, and obtain the following:

(22) $$\frac{\partial \mu }{\partial q_{i}}=\frac{\sqrt{|JJ^{T}|}}{2}trace\left(\begin{array}{c} \frac{\partial (JJ^{T})}{\partial q_{i}}{\left(JJ^{T}\right)}^{-1} \end{array}\right)$$

where i=1,2,⋯,n. Then let H(q) denote the Hessian matrix of the robot, and we obtain the following:

(23) $$\dot{J}=H\left(q\right)\dot{q}$$

(24) $$H_{i}=\frac{\partial J}{\partial q_{i}}$$

where $H_{i}\in R^{6\times n}$ is the *i*-th component of the Hessian matrix. According to the differential kinematics of robots, we have the following simplified form:

(25) $$\frac{\partial \mu }{\partial q_{i}}=\frac{\sqrt{|JJ^{T}|}}{2}trace\left(\begin{array}{c} \left(H_{i}J^{T}+JH_{i}^{T}\right){\left(JJ^{T}\right)}^{-1} \end{array}\right)$$

in which *J* and $H_{i}$ only depend on the current joint angles *q* and i=1,2,⋯,n.

According to the dual-arm motion classification, the manipulability sub-objective is established with different strategies. For an independent motion, we maximize the respective manipulability of each arm, as follows:

(26) $$f_{2}\left(q\right)=-{\left(\begin{array}{c} \frac{d\mu }{dq} \end{array}\right)}^{T}\dot{q}=-\left[\begin{array}{cc} {\left(\begin{array}{c} \frac{d\mu_{l}}{dq_{l}} \end{array}\right)}^{T} & {\left(\begin{array}{c} \frac{d\mu_{r}}{dq_{r}} \end{array}\right)}^{T} \end{array}\right]\left[\begin{array}{c} {\dot{q}}_{l}\\ {\dot{q}}_{r} \end{array}\right]$$

Meanwhile, for a coordinated motion, the dual-arm relative manipulability is optimized as follows:

(27) $$f_{2}\left(q\right)=-{\left(\begin{array}{c} \frac{d\mu }{dq} \end{array}\right)}^{T}\dot{q}=-{\left(\begin{array}{c} \frac{d\mu_{rel}}{dq} \end{array}\right)}^{T}\dot{q}$$

Let $x={\left[{\dot{q}}^{T}\;\delta^{T}\;\eta^{T}\right]}^{T}$ be the augmented optimization variable. Then the objective function f(x) can be rewritten as

(28) $$\begin{array}{cc} f(x) & =\frac{1}{2}\left(\lambda_{q}{\dot{q}}^{T}W_{q}\dot{q}+\lambda_{\delta }\delta^{T}W_{\delta }\delta +\lambda_{\eta }\eta^{T}W_{\eta }\eta \right)-\lambda_{\mu }{\left(\begin{array}{c} \frac{d\mu }{dq} \end{array}\right)}^{T}\dot{q} \end{array}$$

(29) $$\begin{array}{cc} \; & =\frac{1}{2}x^{T}\left[\begin{array}{ccc} \lambda_{q}W_{q} & \; & *\\ \; & \lambda_{\delta }W_{\delta } & \\ {}* & \; & \lambda_{\eta }W_{\eta } \end{array}\right]x+\left[\begin{array}{ccc} -\lambda_{\mu }{\left(\begin{array}{c} \frac{d\mu }{dq} \end{array}\right)}^{T} & 0 & 0 \end{array}\right]x \end{array}$$

We use the weight matrixes $W_{\delta }$ and $W_{\eta }\in R^{6\times 6}$ to adjust the magnitude differences between translation and rotation velocities.

## 3. Results

### 3.1. Verification of MVD and Global Planner

**Figure 7:**
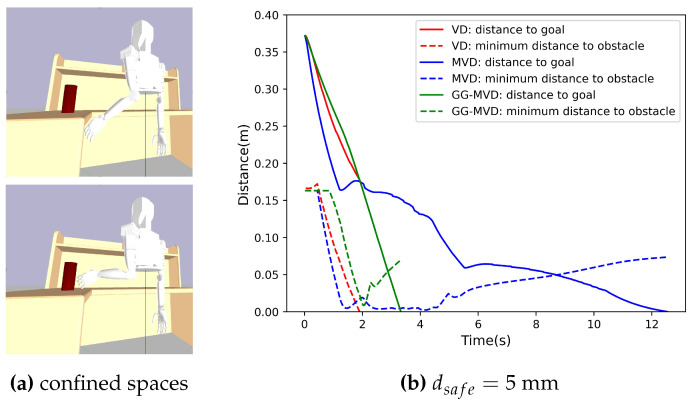
Comparison of different collision avoidance methods in a confined workspace. (**a**) Environment of the picking task. Robot hand is in close proximity to the obstacle when reaching a target grasp pose. (**b**) Minimum Euclidean distances between the robot and the obstacle or the goal pose comparing VD, MVD, and GG-MVD. The VD method in red color failed at 1.92 s. MVD in blue color succeeded at 12.41 s and had a minimum distance of 4.8 mm (to the obstacle). GG-MVD in green color also succeeded and finished around 3.7× faster than MVD at 3.32 s with a larger minimum distance of 8.7 mm (to the obstacle).

To test the validity of the MVD method and global planner module, we have designed an object-picking task with Pybullet. This task simulates a desktop cleaning scene in which the humanoid robot is in close proximity to obstacles while avoiding collision. This requires a highly accurate algorithm for collision avoidance, especially for the robot hand area. Figure 7a shows that the robot hand goes through very confined spaces to reach the target object.

We make a comparison among VD, MVD, and GG-MVD (MVD with global guidance). The first two methods only employ the local reactive controller module, which is set to the independent mode. The arm motion is driven by EE velocity, whose direction is defined by the difference of the current EE pose and the target grasp pose. The translation and rotation components are as follows:

(30) $$\begin{array}{c} v_{dir}=\left(p_{target}-p_{current}\right)/\left|p_{target}-p_{current}\right|\\ \omega_{dir}=Axis\left(R_{target}\cdot R_{current}^{-1}\right) \end{array}$$

where $Axis\left(\cdot \right):R^{3\times 3}\to R^{3}$ is a function mapping a rotation matrix to the corresponding rotation axis. As for the GG-MVD method, the direction is determined by reference waypoints obtained from a global planner. For a fair comparison, the magnitudes of EE velocity are set to be the same. In this experiment, we choose ||v||=0.2 m/s and ||ω||=1 rad/s. Figure 7b illustrates the experiment result when a small safe distance $d_{safe}=5$ mm is adopted. Solid lines represent distances between EE and the goal pose, while dashed lines represent the minimum distances between the robot and the obstacle. The VD method failed at 1.92 s with the robot hand colliding with the desk. This is because the small interactive distance required in a confined environment cannot provide enough safe margin for VD constraints. The MVD method, however, succeeded without collision even though the robot was in an extremely close distance to the obstacle at 1.36 s∼4.63 s. It demonstrates the effectiveness of the stricter rotation constraints defined by MVD. When the distance between two convex geometries is equal to or less than the safety distance, these constraints propel that the witness points on each object remain the same in the next time step. Consequently, there is barely any penetration to the collision safe boundary. Notably, it takes only 3.32 s for the GG-MVD method to finish this task, almost 3.7× faster than MVD. With the full task horizon, the global planner exploits a feasible workspace and generates a more efficient reference trajectory. This trajectory not only avoids the area where low-speed motion or a local minimum occurs due to obstacle avoidance, but also exhibits a shorter overall length.

### 3.2. The Pick–Handover–Place Task

Desktop item organizing is a composite sequence of multiple dual-arm tasks. We define it as a pick–handover–place procedure. In the first part, one arm grasps the target object. Then this object is transferred to another arm in the second part, which is named as self-handover. Then in the final part, the object is placed at the goal pose. The dual-arm motion in the self-handover part is coordinated, while the rest of the time is independent. Our GGDRC is applied to the entire procedure.

In independent mode, the global planner generates the reference trajectory of the EE (hand) region. Meanwhile, in coordinated mode, the planner first generates the reference trajectory of the transferred object, and then converts it to the EE trajectory according to the corresponding grasp relation between the hand and the object. Considering the range of robot workspace, we choose the number of waypoints as k=20 and the maximum step $S_{max}$ based on the start and goal pose and *k*. The preferred safe distance $d_{safe}$ is set to 0.1 m, and the collision penalty coefficient $\lambda_{p}$ to 10. The initial global reference trajectory is given by the interpolation from the start point to the added middle path point and, finally, to the goal point. The central point of the overlapped workspace of two arms is adopted as the middle point. This initialization is effective for most cases. Other parameters in Algorithm 1 are $\epsilon_{f}=0.75$, $\epsilon_{x}=0.005$, α=1.2, and β=0.8.

Accurate environment collision and robot self-collision avoidance throughout the procedure are rigorously satisfied with the MVD method. The translation and rotation coefficient are set as $\xi_{v}=0.8$ and $\xi_{\omega }=pi/3$. For a specific object, we choose a fixed relative grasp pose for both hands at the handover point. Then the relative EE velocity in coordinated constraints (Equation (11)) is determined by the current relative EE pose and goal relative grasp pose in a similar way as in Equation (30). The parameters of the optimization objective (29) for the local controller are $\lambda_{q}=0.01$, $\lambda_{\delta }=50$, $\lambda_{\eta }=30$, and $\lambda_{\mu }=0.01$. We use a large weight coefficient for slack variables to encourage a dual-arm robot to move along with the globally planned path.

The two modules of GGDRC work in a parallel but asynchronous manner. Our object-oriented global planner is implemented in Python and takes only 0.67 s to generate a global reference trajectory in this task. Meanwhile, for the conventional trajectory optimization method, it costs 6.1 s to plan a similar 18-DOF motion even implemented with C++ [18]. Our global planner has made a reasonable dimension reduction and greatly improves the computational efficiency, which satisfies the online re-planning requirement. Considering the low necessity of a high updating frequency of the global guidance, the global planner runs at a lower frequency (1 Hz). The local controller, however, runs in a higher frequency (100 Hz) to ensure that all task constraints are satisfied reactively.

**Figure 8:**
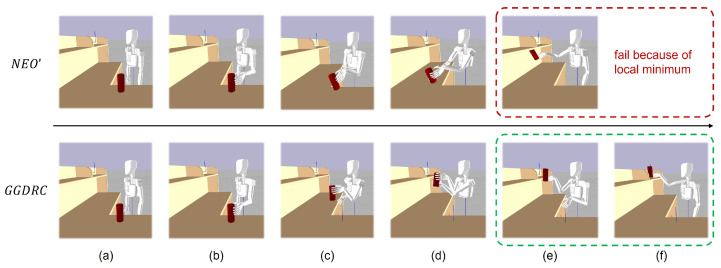
Different motions of a dual-arm robot generated by NEO’ and GGDRC in the pick–handover–place task: (**a**) start configuration, (**b**) object grasped by the left hand, (**c**) two hands approaching each other, (**d**) object self-handover, (**e**) dual-arm independent motion, and (**f**) object placed on the shelf. NEO’ failed in this task because of the local minimum, while GGDRC succeeded with the global reference trajectory of the object.

**Figure 9:**
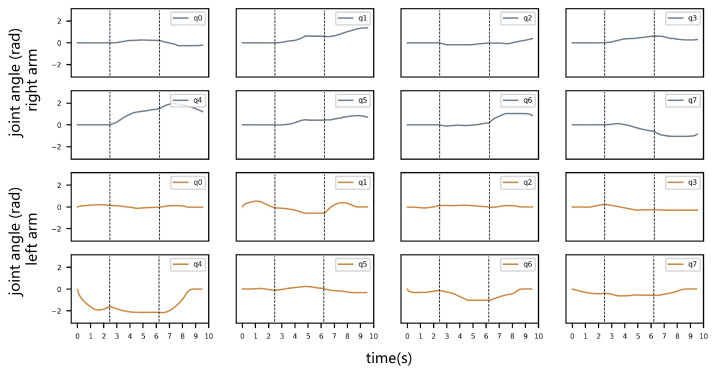
Joint angles of the whole pick–handover–place process with two arms plotted separately. There are two vertical dashed lines in every subplot. The first line represents the moment when the left robot hand grasps the object; in the meanwhile, the global planner starts to generate the reference trajectory of the object. The second one denotes that self-handover takes place, and the object is transferred from the left hand to the right hand.

We compare our proposed framework, GGDRC, with NEO’ [23] (NEO’ represents NEO + MVD, since the original VD method leads to collision between the robot hand and the environment). Figure 8 shows snapshots from the pick–handover–place task. With NEO’, the main objective in the handover part is to reach the target relative grasp pose, which means that two end effectors move to minimize the pose error along the fastest direction. This short-sighted behavior leads to the handover point close to obstacles, which reduces the flexibility, and also causes the transferred object to get stuck in the local minimum at the picking and placing part. However, with GGDRC, the dual-arm robot has finished this whole procedure and exhibits more human-like motion characteristics. Each joint angle during this task is shown in Figure 9.

**Figure 10:**
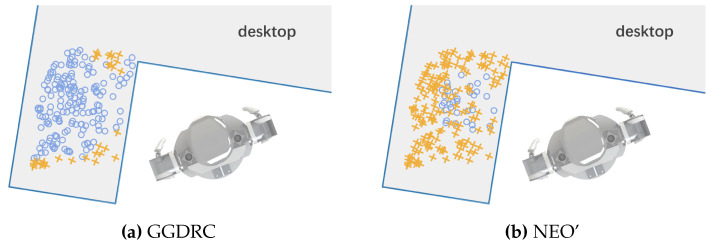
Top view of the repeated pick–handover–place experiment with 200 trials. Randomly sampled start positions are illustrated in the left area of the desktop. Blue circle markers denote the successful trials, while orange cross markers denote the failed trials. (**a**) The result of GGDRC with a success rate of 84.5% and an average runtime of 10.83 s. (**b**) The result of NEO’ with a success rate of 16.5% and an average runtime of 19.43 s.

A repetitive experiment consisting of 200 trials is also conducted to demonstrate the robustness of GGDRC. The start position of the target object is randomly sampled from the left area of the desktop, as shown in Figure 10, followed by the same pick–handover–place procedure. The object is transferred to the same end position. In every trial, two methods (GGDRC and NEO’) are both tested. Any collision or runtime exceeding 25 s is assumed as failure.

Except for some fringe area, which is nearly out of the left arm workspace or contains distinct non-convex geometric features, GGDRC is able to complete this task in a reliable manner and presents a 84.5% success rate in 200 trials. The average runtime for success trials is 10.83 s. However, NEO’ is easily trapped in the local minimum and only has a success rate of 16.5% with an average runtime of 19.43 s. Key results are summarized in Table 1. NEO’ tends to take more time because it uses a straight approaching strategy from the robot EE to target a pose and adopts a simple retreat heuristic when the environment obstacle blocks its moving path. Meanwhile, in our proposed framework, the global planner can effectively analyze environment information, provide forward-looking guidance, and be free from the local minimum. Meanwhile, the local controller ensures accurate obstacle avoidance and coordinated dual-arm motion.

### 3.3. Dynamic Environment Experiment

In the last experiment, we use dynamic scenarios containing a moving obstacle to test the reactive performance of GGDRC. There are two groups of experimental setups in which the moving obstacle will approach the dual-arm robot workspace from either the left or the right side. These arrangements are designed to simulate human disturbance that may occur in a home organizing environment. The humanoid robot still performs the pick–handover–place task in this experiment, but without the dynamic collision avoidance, the moving obstacle will collide with the robot’s hand region during the handover portion. The obstacle is modeled as a sphere with a diameter of 0.05 m, and set to different velocity levels. We use an incremental velocity sequence from 0.1 m/s to 1 m/s with 0.1 m/s intervals to test GGDRC. Additionally, there are 15 trials for every velocity level. The trajectory of the moving obstacle starts from position $p_{0}\in R^{3}$ to position $p_{1}\in R^{3}$ and then back to $p_{0}$ with a trapezoidal speed profile. At every trial, $p_{1}$ is randomly sampled from a sphere region with a 0.1 m radius where collision will happen if no action is taken.

We adopt an autonomous switching strategy for the modification of parameters in a local reactive controller. If a moving obstacle is detected, a series of modifications will be implemented. On the one hand, the weight coefficients in the objective function of the local optimization are changed. The weights of the two slack variables are reduced to lower the influence of global guidance since dodging the moving obstacle is the top priority. Additionally, the weight of manipulability is increased to ensure that joint velocities remain within the physical limits when the robot needs to avoid obstacles at high speeds. Therefore, we choose $\lambda_{\delta }=0.01$, $\lambda_{\eta }=0.01$, and $\lambda_{\mu }=0.05$. On the other hand, the collision check distance $d_{check}$ and the safe distance $d_{safe}$ of MVD are increased, taking into account that avoiding moving objects requires a certain reaction time. Additionally, unlike static manipulation, dynamic obstacle avoidance prefers less fine-grained motion modeling but a more pre-reserved safety margin. Hence, we choose $d_{check}=0.3$ m and $d_{safe}=0.1$ m.

**Figure 11:**
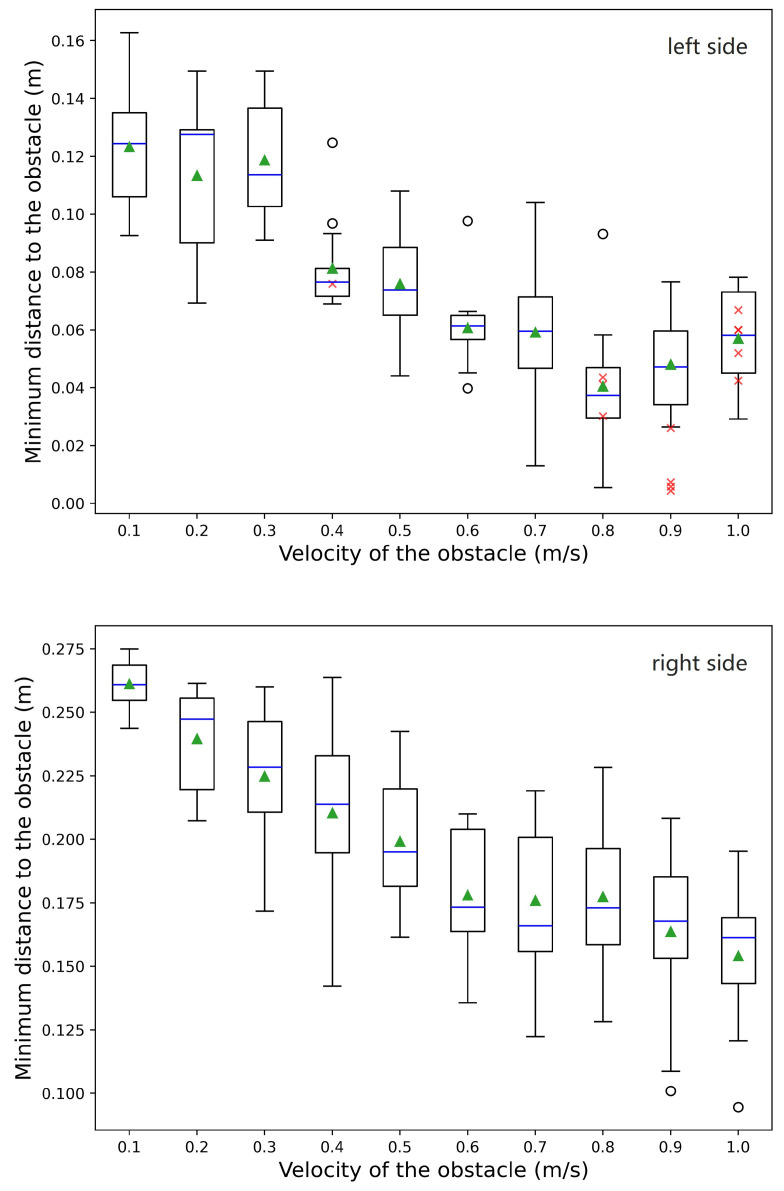
Distribution of minimum distance $d_{min}$ from the robot to the moving obstacle with different velocities. Obstacle approaching (**Top**) from the left side; and (**Bottom**) from the right side. For each value of velocity, a boxplot shows the distribution of 15 data points. The upper and lower whiskers are based on a 1.5 IQR value, and outliers are indicated by hollow circle markers. The blue middle line across the box denotes the median value, and the green triangle denotes the arithmetic mean value of valid trials. In addition, the red cross markers represent failed trials because of collision.

Table 2 the shows results of the dynamic experiment. When the obstacle approaches from the right side, GGDRC achieves a 100% success rate at all velocity levels. For the left side, the results are still promising when the obstacle velocity is less than 0.7 m/s, but gradually decrease to 67%. Due to the confined area surrounded by the desk and robot torso, the feasible range of left arm configuration is limited in the handover portion, which makes it harder to dodge fast-moving obstacles. Figure 11 demonstrates the distribution of minimum distance $d_{min}$ from the robot to moving obstacles during the experiment. Generally speaking, with the increasing velocity of moving obstacles, the minimum distance during the task gradually reduces as less time is left for the local controller to respond. In the right-approaching scenarios, all the average $d_{min}$ is greater than 0.15 m, which means no penetration to the safety margin specified by MVD. For the left side, although it is not able to always satisfy the minimum distance expectation, GGDRC still accomplishes a task success rate of 98.6% in the velocity interval from 0.4 m/s to 0.7 m/s. Additionally, we find that the average minimum distances of the succeeded trials with speeds greater than 0.7 m/s are basically around 0.04 m. All these results demonstrate that our GGDRC controller is practical with dynamic obstacles, especially when their velocities are less than 0.7 m/s.

## 4. Discussion and Conclusions

This article presents a globally guided dual-arm reactive motion controller for humanoid robots performing desktop organizing tasks in an unstructured domestic environment. Within this method, the global planner module overviews environment information, generates object-oriented reference trajectory in a task space, and provides feasible guidance for the dual-arm motion. The time consumption greatly decreases because of the dimension reduction of the optimization problem. Our QP-based local reactive controller module achieves dynamic collision avoidance with the modified velocity damper method and meets the requirement of dual-arm coordination constraints utilizing robot relative Jacobian. It also takes advantage of robot redundancy and optimizes manipulability to cope with complex manipulation tasks. Results demonstrate that our method shows accurate collision avoidance precision and success rate in a motion-restricted and dynamic environment.

GGDRC bridges the gap between global planning and reactive methods for dual-arm manipulations. It highlights the guidance rule of a global trajectory planner and the real-time performance of local reactive controllers. Our method improves the computational efficiency in high-dimensional planning problems while avoiding the local minimum in traditional reactive methods, and provides an alternative framework for the cooperative motion control of dual-arm robots. This contributes to the realization of future humanoid robots performing complex bimanual cooperative tasks in unstructured and dynamic environments.

One of the limitations of our work is that GGDRC contains a few hyperparameters requiring manual adjustment. A dynamic parameter tuning strategy based on environment information will be beneficial to the robustness. As this method only adopts a fixed envelope grasping gesture, future studies could design a cooperative grasp synthesis for dexterous hands and generalize GGDRC to different bimanual manipulation tasks. Additionally, a more significant representation of an environment incorporating object affordance and dynamic motion prediction is another direction for extensions.

## Figures and Tables

**Table 1:** Results of the repetitive experiment with 200 trials. Time consumption is calculated from the start of the trajectory planning to the end of the task execution; path length is calculated from the total movement of the object.

	GGGDRC	NEO’
Success rate	84.5%	16.5%
Time consumption (s)	10.83	19.43
Path length (m)	1.244	1.119

**Table 2:** Evaluation of GGDRC in a dynamic environment using two metrics: (a) success rate of 15 trials and (b) average minimum distance to a dynamic obstacle and the standard deviation. The obstacle moves in ten velocity levels from 0.1 m/s to 1 m/s from each side of the robot.

Velocity (m/s)	Right Side		Left Side	
	Success Rate (%)	Avg. $d_{\min}$(m)	Success Rate (%)	Avg. $d_{\min}$(m)
0.1	100	0.261 ± 0.010	100	0.123 ± 0.021
0.2	100	0.240 ± 0.019	100	0.113 ± 0.026
0.3	100	0.225 ± 0.027	100	0.119 ± 0.019
0.4	100	0.210 ± 0.035	93.3	0.081 ± 0.015
0.5	100	0.199 ± 0.023	100	0.076 ± 0.018
0.6	100	0.178 ± 0.022	100	0.061 ± 0.013
0.7	100	0.176 ± 0.029	100	0.059 ± 0.022
0.8	100	0.177 ± 0.028	80.0	0.040 ± 0.021
0.9	100	0.164 ± 0.030	73.3	0.048 ± 0.017
1.0	100	0.154 ± 0.026	66.7	0.057 ± 0.016

## Data Availability

The data and code of the current study can be obtained from the authors upon reasonable request.
